# Chromogranin A and Its Fragments in the Critically Ill: An Expanding Domain of Interest for Better Care

**DOI:** 10.3390/pharmaceutics14102178

**Published:** 2022-10-12

**Authors:** Francis Schneider, Raphaël Clère-Jehl, Francesco Scavello, Thierry Lavigne, Angelo Corti, Tommaso Angelone, Youssef Haïkel, Philippe Lavalle

**Affiliations:** 1 Médecine Intensive—Réanimation, Hôpital de Hautepierre, Hôpitaux Universitaires de Strasbourg, Faculté de Médecine, FMTS at Unistra, 67085 Strasbourg, France; 2 Biomaterials and Bioengeneering, UMR_S1121, FMTS at Unistra, 67085 Strasbourg, France; 3 IRCCS Humanitas Research Hospital, 20089 Rozzano, MI, Italy; 4 Hygiène Hospitalière et Médecine Préventive, Pôle de Santé Publique, Hôpitaux Universitaires de Strasbourg, 67091 Strasbourg, France; 5 San Raffaele Scientific Institute, Division of Experimental Oncology, Vita-Salute San Raffaele University, Via Olgettina 58, 20132 Milan, MI, Italy; 6 Laboratory of Cellular and Molecular Cardiac Pathophysiology, Department of Biology, Ecology, and Earth Science, University of Calabria, 87036 Rende, CS, Italy; 7 Nouvel Hôpital Civil, Hôpitaux Universitaires de Strasbourg, Faculté de Chirurgie dentaire, Université de Strasbourg, 67091 Strasbourg, France

**Keywords:** albumin, biomaterials, Catestatin, Chromogranin A, Chromofungin, critically ill, outcome, prognosis, superbugs, Vasostatin-I

## Abstract

Life-threatening diseases challenge immunity with a release of chromogranins. This report focuses on Chromogranin A (CGA) and some of its derived peptides in critically ill patients, with attention paid to their potential to become biomarkers of severity and actors of defense. First, we studied whether circulating CGA may be a biomarker of outcome in non-selected critically ill patients: CGA concentrations were reliably associated with short-term death, systemic inflammation, and multiple organ failure. Additionally, when studying Vasostatin-I, the major N-terminal fragment of CGA, we noted its reliable prognostic value as early as admission if associated with age and lactate. In trauma patients, CGA concentrations heralded the occurrence of care-related infections. This was associated with an in vitro inhibitor impact of Chromofungin on both NF-kappa B- and API-transcriptional activities. Secondly, in life-threatening disease-induced oxidative stress, the multimerization of Vasostatin-I occurs with the loss of its anti-microbial properties ex vivo. In vivo, a 4%-concentration of non-oxidized albumin infusion reversed multimerization with a decrease in care-related infections. Finally, in vitro Catestatin impacted the polymorphonuclear cells-Ca++-dependent, calmodulin–regulated iPLA2 pathway by releasing immunity-related proteins. Furthermore, human Cateslytin, the active domain of Catestatin, helped destroy S. aureus: this prompted the creation of synthetic D-stereoisomer of CGA-derived peptides against superbugs for the protection of implanted devices. In conclusion, CGA consideration in the critically ill is only starting, but it offers interesting perspectives for improved outcomes.

## 1. Introduction

**Figure 1:**
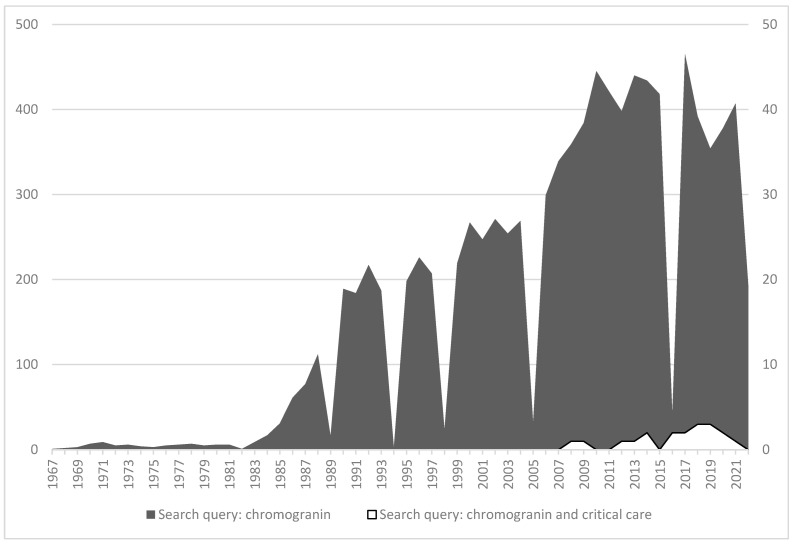
Number of annual papers issued in the PubMed database according to the query from 1967 to 2022. Please note that the scale of measurement is ten times larger for the query “Chromogranin” (left vertical axis) than for the query “chromogranin and critical care” (right vertical axis).

Chromogranins proteins had attracted the interest of scientists and clinicians since the mid-sixties when Blaschko H et al. [1] and Helle KB [2] released the first two manuscripts stored in the PubMed database on this topic. Since then, no less than 9891 papers have been published on chromogranins, of which, however, only 19 are indicated as concerning data recorded in patients admitted in critical care wards (Figure 1). The interest in chromogranins as mediators of life-stressing diseases on human health, although thus only at an early stage, has been slowly growing over the last decade. Fifteen years ago, our study group was among the first to consider CGA as a possible object of examination in critical illness. Yet, this area of assessment is complicated. First, in vivo studies require testing large cohorts of patients with clinical phenotypes as similar as possible. This necessitates extensive multicenter studies when clinically relevant hypotheses have been validated in feasibility studies. For instance, now that we have identified Vasostatin-I (VS-I) as a myocardial depressant factor [3], it becomes interesting to gather data to comprehend its role in depressed cardiac contractility in acute hypokinetic shock. Second, clinicians must test in vivo clear-cut null hypotheses, which may be very difficult in multiple organ failure patients. As an example of such difficulty, systemic inflammation in response to strong pro-inflammatory triggers is frequent in intensive care units (ICUs), but in vivo studies require the assessment of chromogranins at the same stage of a given disease mandatorily. In each setting, pharmacokinetics, pharmacodynamics, and the volume of distribution of the proteins of interest are not currently established; and this is independent of the availability of adequate techniques of measurements that may vary according to the characteristics of both the test used and the physiological liquids from which samples have been harvested. Finally, having decided to assess the presence of these proteins, adequate biological sampling techniques must be chosen. This sometimes requires the in-house development of biological assays [4].

Patients admitted to an ICU for a disease with organ failure are at risk of short-term death. In this setting, in response to the initial assault on their integrity, they develop a systemic inflammatory response to maintain vital organs functioning and optimal tissue repair. This response is temporary and well-balanced between pro- and anti-inflammatory mediators; it results in recovery, provided the initial trigger of the disease does not overwhelm physiological responses. Following initial local injury, the organism develops (i) a hormonal, metabolic response, (ii) an immunological response with a neurological component, and (iii) a hemodynamic response called “acute circulatory failure” or shock [5,6]. During that period, the organism rapidly releases chromogranins not only from adrenal medulla stockpiles but also from the diffuse neuroendocrine cell islets existing throughout the organism. From blood concentration assessments, physicians can indirectly assess the balance between synthesis, release, and clearance of the molecules while also considering the possible impact of extra-renal purification techniques.

Bearing these precautions in mind, we report our experience on CGA (and related peptides) in critically ill and experimental conditions related to acute and severe illnesses.

## 2. CGA and Vasostatin-I (CGA1-76) Are Biomarkers of Severity in the Critically Ill

In 2007, we assessed whether CGA could become a biomarker of severity in life-threatening diseases on hospital admission. Our primary aim was to improve the triage of intermediate-severity patients to select the most adapted care for them. At that time, the agreed practice was to assess severity in ICU patients with the Simplified Acute Physiology Score 2 (SAPS II) based on a European/North American multicenter study [7]. This score provides an accurate estimate of the risk of death in the ICU without having to specify a primary diagnosis. It proved efficient notwithstanding that since it was available only at hour 24 of admission, it could not become a tool for early triage, whereas a rapidly available and performant biological test would.

The idea of assessing CGA was prompted by the fact that life-threatening diseases trigger a physiological response to an aggressive challenge to health with a triple component: (i) a hormonal, metabolic response; (ii) an immunological response, and (iii) a hemodynamic response—all of which are also linked by a neural immunity balance [6]. Following damage to the body’s integrity, a patient will activate the release of various hormones, such as adrenalin and cortisol, as well as many others [8]. The adrenocortical response is similar in all mammals: afferent impulses from receptors implanted in the injury site stimulate the secretion of hypothalamic-releasing factors, further stimulating the pituitary gland. Consequently, the adrenal cortex releases cortisol and the adrenal medulla adrenaline together with chromogranins that are stored and co-released by the chromaffin cells. A feasibility study confirmed the hypothesis that patients with acute organ failure present at admission increased serum concentrations of CGA as a probable component of the early hormonal “fight-or-flight” adaptive response to stress [9]. Importantly, circulating CGA concentrations increased in association with systemic inflammation rather than with infection and the SAPS II, suggesting a possible link with either survival or one of the components of the score. These data align with what happens in humans even in the context of a stressing injury less severe than trauma, thus indicating that the slightest injury to the skin integrity also results in a signal of stress with the release of chromogranin-derived anti-microbial peptides (AMP) [10]. In a confirmation study [11], we reported that CGA concentrations correlate better with systemic inflammation (assessed by both C-reactive protein and procalcitonin) than with infection. Yet, CGA levels reached the highest values in septic shock. In addition, admission CGA values were equivalent to the SAPS II in predicting 28-day mortality. This offered the attractive opportunity for assessing outcomes as early as a few hours after admission in patients free of any other cause of CGA increase.

Further insight into the use of CGA as a biomarker of triage required an extension of the study size. We wondered whether a single admission dosage of VS-I (CGA-1-76), the major N-terminal fragment, would enable greater accuracy than a single CGA test. In a pilot prospective observational study performed in third-level French ICUs, we demonstrated that admission concentrations of VS-I were increased when compared with the values recorded in controls, and this was even more so when the shock was present [12]: VS-I concentrations above 3.97 ng/mL were indicative of poor outcome. Furthermore, including arterial lactate and age in the prediction model improved the reliability of the assessment, making it significantly better than the SAPS II as early as 4 h after admission.

In a further study, we investigated whether CGA could be a marker of the severity of the initial challenging disease as regards its impact on morbidity. Care-related infections are a major issue in critical care for many reasons (costs, bed- blocking and emergence of antibiotics-resistant superbugs). We chose the study phenotype of “multiple trauma” in patients previously in good health. This model appeared more suitable for investigating the impact of a basic injury on health, given that older patients with comorbidities were excluded. The study model, by definition, included a “two-hit” challenge to health: first, the trauma itself and, shortly thereafter, the surgical procedure, which represents a second hit for CGA release. In such patients, we noted an altered plasmatic CGA response revealing a potential mechanism for an association with nosocomial infection [13]. Over several days from admission, CGA concentrations increased compared with control values, but they also leveled off at a relatively high level associated with acute renal failure. Importantly, admission values of CGA significantly increased in those multiple trauma patients who subsequently developed a nosocomial infection: a concentration of 67 ng/mL predicted this occurrence with a sensitivity of 100% and a specificity of 70%, leading to a negative likelihood ratio of almost zero. Recently, we confirmed that admission CGA achieved similar performance for predicting nosocomial infections in COVID patients requiring oxygenation support [14]. This suggests that CGA may reflect not only the triggering disease but also the neuro-hormonal response by the neuroendocrine tissues. In multiple trauma, we finally investigated the ability of VS-I to modulate the innate response of monocytes that are called on to upgrade the patient’s defense [13]. Acting as a cell-penetrating peptide, the CGA47-70 fragment, not however including either its scrambled or its prolonged isoforms, was able to downregulate both NF-kappa B and AP-1: this suggests an indirect anti-inflammatory pathway potentially entailing a risk of temporary immune deficiency. These data support the possible occurrence of some forms of care-related infections when proteases of CGA are upregulated.

## 3. Fine-Tuned Albumin Infusion Modifies CGA-Derived Peptides Multimers In Vitro and Impacts on Nosocomial Infection Occurrence

**Figure 2:**
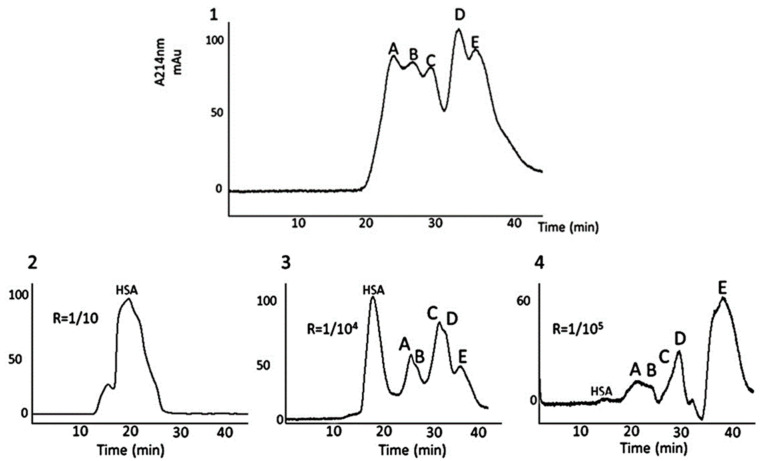
Gel filtration HPLC of purified and oxidized VS-I samples to evaluate the impact of different concentrations of therapeutic human serum albumin (HSA) on multimers of VS-I. For the four graphs, the X-axis corresponds to the elution time (expressed in min), which is linearly related to the molecular weight of the components included in the peaks. The Y-axis corresponds to absorbance expressed in milliUnits of absorbance. Whether in vitro or in vivo, the oxidation of VS-I leads to multimerization, as shown on the first chromatogram (upper graph, numbered 1). The monomeric form of the peptide corresponds to peak E, while peaks A-D correspond to VS-I multimers. When adding fresh, non-oxidized therapeutic HSA at a molar albumin/VS-I ratio (R) from 1/10 to 1/10^5^ (as shown on chromatograms 2–4), the release of the monomeric VS-I increases (see changes in the amplitude of peak E). This counterintuitive result explains the possible release of monomers of VS-I with the restoration of its anti-microbial properties [15].

**Figure 3:**
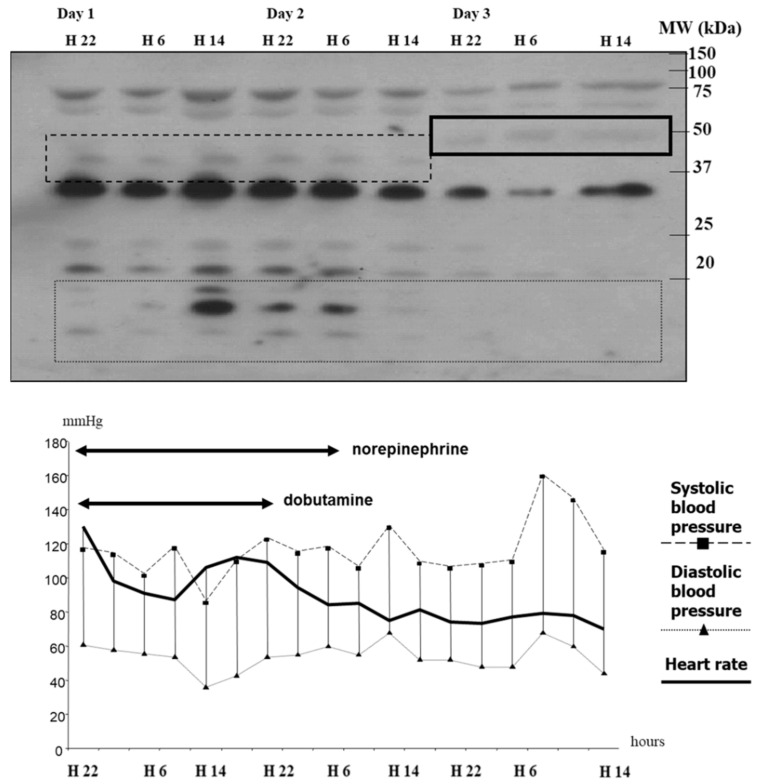
Typical Western blotting analyses of plasma samples to evaluate the time-dependent changes in extracellular processed VS-I (**top** panel), and simultaneous hemodynamic profile (**bottom** panel) during a human septic shock. Time (Day X) runs over 3 days from admission (Day 1, hour 10:00 pm (H22)) to the end of the third day (Day 3, 02.00 pm (H14) and is represented on the X-axis (top and bottom lines). Plasma samples harvested by 8 h, from admission until Day 3, were immediately centrifugated (4000 rounds/min at 4 °C), and SDS-PAGE electrophoresis followed by electrophoretic blotting with immunological detection of VS-I was performed in standard conditions. The anti-VS-I antibodies were a generous gift of Pr A. Corti, Milan, Italy. Periods of norepinephrine and dobutamine infusion are represented as bold arrows from admission until weaning. Please note that the processing of chromogranin A is notably changing around Day 2 at 2:00 (H14) when the patient’s circulatory status no longer requires the two vasopressors. The global immunoblots are decreasing in intensity, and both small and large molecule multimerization is decaying (top panel). This phenomenon is contemporary to the decrease in doses of vasopressors (norepinephrine and dobutamine) and corresponds to circulatory failure recovery. Altogether, these data explain (i) why dosages of any of these proteins must be performed at a similar time window of the disease if a proper interpretation of their role is to be considered; (ii) that a pharmacological intervention must be scheduled at a moment when it can be efficient. Thus, our data on 4% albumin-impact on multimerization show that such an intervention must start as early as possible after admission, and it has no sense after the 5th day of disease onset [16]. Please remember that the apparent molecular weight (MW) of full-length CGA is around 70–75 kDa, and that of VS-I is approximately 18 kDa, which explains the immunoblotting of multimers on the top panel. Boxes (solid line, dashed line…) focus on processed molecules of interest, tagged with VS-I-antibodies: a careful identification must specify whether some multimers are not just large monomeric, full-length CGA molecules containing the VS-I domain.

Care-related infections are a matter of worry in the ICU: they increase the costs of treatment and length of stay and kill, on average, 10 to 15% of the patients. Finally, acute stress and anti-microbial treatments are also responsible for the emergence of transmittable-resistant microbes. Despite hygiene, antibiotics stewardship, and precautions, these infections will occur in many patients with systemic inflammatory conditions at the acute phase of a disease. Oxidative stress induces damage to proteins within the circulation and beyond. The mechanism of oxidative stress undoubtedly affects many proteins, including those belonging to innate defense. In several patients, we recorded multimers of interest at the acute phase of the disease, including multimers of granins (see Figure 2 and Figure 3). These multimers persist longer in the circulation of those patients with the highest and longest rate of infusion of norepinephrine for shock. Human serum albumin (HSA) displays properties for the care of critically ill patients. Among others, HSA provides an opportunity to restore in vitro the native status of proteins, as reported previously by our group [15]. However, this effect has never been reported in ICU patients that are prone to develop either colonization or infection. We decided to perform a pilot study on critically ill patients at risk of nosocomial infection to test whether therapeutic non-oxidized HSA would prevent such infections [16]. This study included a biochemical analysis of the interactions of the CGA-derived peptide VS-I, for which the anti-microbial activity is related to its non-oxidative state [15]. The results were that: (i) in vivo, therapeutic HSA significantly lessens both colonization and infection occurrences in patients with shock; (ii) this was possible provided therapeutic HSA is prescribed as a continuous low dose infusion of 4% HSA. We showed, in addition, that, in vitro, both natural and recombinant VS-I develop biochemical interactions with several natural and synthetic isoforms of albumin (HSA, bovine serum albumin, therapeutic HSA) via the hydrophobic domain of VS-I17-40 (which includes the disulfide bridge C17-C38). This allows the oxidation of VS-I to be reversed, rendering it more efficient as an anti-microbial protein even in tissues where the pH decreases at 6 in microcirculation during shock. We deduced that the rate of therapeutic HSA infusion is essential in vivo when seeking to restore the physiologic activities of defense. A prospective multicenter open-label randomized trial confirmed this data in septic shock patients for which continuously infused 4% therapeutic HSA over the first week of shock decreased nosocomial infection by two-thirds when compared with the intermittent infusion of similar doses of 20% HSA [17]. These results are noteworthy because they explain the discrepancies existing in meta-analyses on the benefit of therapeutic HSA: many studies postulate the lack of efficiency of therapeutic albumin in septic conditions, whereas others have found significant improvements in restricted populations [18,19]. In fact, protocols of infusion and amounts of therapeutic albumin differ from one study to another, and so do the isoforms of albumin tested, which explains why physicians do not achieve the goal of defense reversion. Our final proposition is that therapeutic albumin should be: (i) chosen as 4% albumin with a high potential of antioxidant activity [20]; (ii) infused continuously at a rate of 10–12 mL/kg/24 h over 5 days to limit the risk of care-related infections.

## 4. In Vitro, CGA-Derived Peptides Modify the Immunological Activities of Specific Cells Belonging to Defense

CGA has a prohormone function; numerous cleavage products of this protein display activity in the domain of defense [21,22]. Therefore, we speculated that the CGA-derived- peptides Chromofungin (CHR, CGA47-66) and Catestatin (CAT, CGA344-364) might impact the functioning of human cells involved in immunity. We harvested polymorphonuclear cells (PMNs) and monocytes from healthy controls. We exposed them ex vivo to the peptides to assess the consequences as they may occur in vivo during systemic inflammation.

We first performed experiments on PMNs [23]. We demonstrated that, after intracellular penetration, both CHR and CAT provoked a rapid and synergic Ca++- entry with no lytic effect on the cells. This occurred provided free calcium was available in the extracellular space. It was concentration-dependent and in the range of concentrations relevant for clinical effects. We also explored the impact of scrambled isomers and amino-acid substitutions. In doing so, we identified the need for a perfect respect of the chemical primary structure of both CAT and CHR to obtain the expected pharmacodynamical impact, indicating that a precise mechanism of both cell entry and action is required for physiological effects. The Ca++- entry evoked by CHR and CAT is consistent with Ca++-selective store-operated calcium channel activity. Once inside the cells, CAT and CHR interact with calmodulin, thereby allowing the release of lysophospholipids by membrane-bound iPLA2 and subsequent store-operated calcium channels. As an ultimate result of intracellular Ca++ concentration increase, the PMNs release secretions, among which we isolated factors involved in innate immunity such as lactotransferrin, neutrophil gelatinase, lysozyme, S100 A, and S100B calcium-binding proteins. These data point to a role of CAT and CHR in Ca++ signaling outside the chromaffin cells, with an impact on the activation of PMNs through a mechanism not related to a cellular membrane-bound receptor but in line with a cell-penetrating peptide activity. This action explains how the neuro-hormonal response to stress may trigger a rapid-onset effective enhanced pro-inflammatory PMNs-related mechanism of defense in vivo in any vascularized tissue where an insult occurs. It is also of note that CAT has been reported to act via the nicotinic acetylcholine receptor (nAChR), a classical surface receptor, which can also participate in anti-inflammatory responses through neural immunity regulation [6].

Our group also explored monocytes. We assessed a possible effect of the CGA47-70-derived peptide (which includes CHR) detected in the plasma of multiple trauma patients who are prone to develop care-related infections [13]. This molecule entered the monocyte progressively over 5 to 15 min with at least two intracytoplasmic localizations, one of which was detected in the perinuclear region. In further cellular investigations, including luciferase assays, we showed that CGA47-66 could inhibit both NF-kappa B and AP-1, which play a role in amplifying and perpetuating the inflammatory processes in vivo. Such activities suggest an anti-inflammatory potential for this peptide with a risk of deleterious imbalance of innate immunity.

## 5. CGA-Derived Peptides as Actors against Superbugs

Antibiotic-resistant microbes (bacteria, fungi, and yeasts) are detected increasingly in samples harvested from ICU patients, and they trigger significant morbidity when comorbidities are present. There is, therefore, a need for better tools to cure patients, in addition to the discovery of new anti-microbial drugs.

Based on the observation that bacterial host cells genetically engineered to express CGA for industrial production are dying upon the induction of CGA expression, our group hypothesized that chromogranins impact bacterial survival (see in [22]). Following the HPLC of the protein material secreted by chromaffin cells, it was concluded that several CGA-derived peptides and CGA itself, proenkephalin-A, and free ubiquitin participated partially in the struggle for survival after a stressing challenge. A summary of the anti-bacterial, antifungal, and anti-malaria properties of some of these chromaffin cell-derived peptides was given in a recent review [22]. Interestingly, while these properties are related to the CGA-derived molecules, they may sometimes be enhanced through synergic associations with either therapeutic albumin or commercially available anti-microbial drugs. Indeed, since AMPs interact with cell membranes, they represent candidates to potentiate anti-microbial drugs, as shown for some molecules marketed for ICU patients [24,25]. Our data also suggest that, sometimes, infectious diseases occur when these AMPs fail to fulfill their missions in oxidative stressing conditions.

Recently, our group also reported that CTS (CGA344-364), but not Cateslytin (CTL, CGA352-366), interacts with circulating albumin, which underlines the important role of the C-terminal part of CTS for the binding process [26]. This interaction improves the anti-microbial activity of this peptide against C. albicans at a concentration of 4 µM, demonstrating a synergistic effect [26].

Finally, human intervention on some of the L-isomers of these molecules may also modify their potency in an attempt to coat medical implants [24,27]. In recent studies, we have tested natural peptides against superbugs carried by ICU patients. It emerged that some natural and some synthetic peptides, though not all, recovered significant bactericidal activity in vitro when associated with or chemically modified. Investigations further showed that AMPs act as either cell-penetrating peptides destabilizing the cell wall of the microbes or as intracellular molecular actors interacting with calmodulin with subsequent limitation of the rate of activity of calmodulin-activated enzymes, some of which play a role in hyphal growth. These data prompted the conclusion that biomaterials intended for human implantation may benefit from the coating by such molecules or from the latter being sprayed (on wounds, for instance). Indeed, contaminations of medical devices and surgical sites continue to contribute to significant hospital morbidity. We have, therefore, successfully moved to functionalize bioprostheses with biomaterials designed to combat biofilm-associated infections. Interestingly, a self-killing approach with the bacterial-controlled release of AMPs has been reported [28]. Lastly, CGA-derived peptides were included in hydrogels to prevent oral cavity infection [29]. CAT was functionalized with polyarginine and hyaluronic acid on a silver platform: this, in addition to CAT’s anti-microbial activity, strongly limited the local production of inflammatory cytokines. This is a matter of interest in relation to buccal implantation [30], with potential as regards devices requiring transient implantation in ICU patients.

## 6. Enigma and Future for CGA and Its Derived Peptides in the Critically Ill

Based on available data, one first challenge is understanding the mechanisms and relevance of the multimerization of CGA and some of its derived peptides in acute stressing diseases. As shown in Figure 3, according to time from admission to the recovery of shock, we have studied by Western blotting analyses the plasma of septic shock patients with antibodies directed against VS-I. As indicated, we have observed time-dependent changes in the processing of VS-I-tagged multimers in the bloodstream during the first days of ICU stay. These data indicate time-dependent changes in the processing of molecules, which supports characterizing chromogranins (CGA, CGA-related peptides, and chromogranins B and C) as acute phase proteins. The multimers rapidly diminished and, in a second phase, progressively vanished from circulation at the time when the treatment by catecholamines was possible to stop. Whether multimerization is just a modality of transport for CGA from chromaffin cells to distant targets in tissues or a mechanism of protection of CGA from enzymatic processing within circulation in this setting remains unresolved and merits close understanding to avoid interrupting a physiological process intended to protect CGA from immediate endogenous processing. On the one hand, the pharmacological manipulation of multimers with low doses of therapeutic albumin enables the release of monomeric molecules if these molecules are already oxidized [16]. On the other hand, the release of CAT, which has no such disulfide bridge available, requires another pharmacological approach, possibly by limiting the upregulation of inducible enzymes responsible for the processing of CGA. In clinical settings with systemic inflammation, such intervention has been recommended for vascular iNO-synthase inhibition. Finally, there is another challenge in relation to ICU patients as far as health stress is concerned: would CGA predict the risk of re-admission to an ICU for an improving patient when he/she leaves the ICU for an intermediate care facility? This issue has never been reported to date but would be of significant interest in situations of overcrowded ICUs.

A second issue concerns the possible use of CGA-derived peptides with defense properties in helping to keep implantable medical devices immune to infectious attacks once implanted. Such a development would not only be of interest to surgeons who implant several categories of prostheses but would also represent a significant breakthrough for critically ill patients. Although these proteins are endogenous peptides with few detectable side effects at nano- or micro-molar concentrations, convenient therapeutic use in humans has never been found. No data exist on in vivo consequences of the infusion of VS-I to test vasoactivity in humans. However, some experimental ex vivo studies have described the effect of VS-I on human vessels and in experimental animals [31,32,33,34]. Although this peptide has proven capable of ex vivo fungicidal and anti-bacterial activity even in multidrug-resistant microbes, it has never been tested in vivo as an anti-microbial drug. No ethical issues have been reported as explaining such a situation. Still, one can reasonably imagine how large the amount of VS-I required to produce persistent physiological effects on humans would be. One additional explanation is that the efficiency and the cost/effectiveness ratio of the drug used as a single anti-infectious agent would be questionable. Nonetheless, it does not seem unreasonable to test this approach to prevent colonization by superbugs in critical patients as they frequently display certain forms of immune suppression [35] or even as local adjuvant treatment. Duration of the peptide availability for long implantation is another limit for such use. Our group has recently proposed that implantable devices can be coated whenever possible to lessen the risk of care-related infections [27,28]. We have succeeded in incorporating CAT—and its active core CTL—by linking them to materials through a spacer, which is cleavable by enzymes from bacterial strains prone to colonizing intravascular prostheses. This opens the perspective of a new defense strategy for fighting infection of implants: the innovative component of the strategy is that the availability of the AMP is longer and diminishes only if the microbe is present with the required enzyme for the release of the coated peptide. For example, we successfully used the endo-protease Glu-C produced by S. aureus, although it had been previously shown that hyaluronidase from both S. aureus and yeast also works [27,28,30]. To make the strategy even more efficient, we have also tested D-stereoisomers of some CGA-derived peptides: these isomers proved very stable against enzymatic proteolysis. The preliminary results encourage further investigation. First, the dimeric form of CTL linked by three polyethylene glycols substantially enhances the anti-bacterial activity against S. aureus. In contrast, dimerization was not required to ensure better destruction of C. albicans. Second, the D- CTL peptide displays interesting, enhanced activity against some Gram-negative superbugs relevant as far as ICU patients’ infections are considered. Third, the non-toxic peptide DOPA5T- CTL can be employed as a “self-killing strategy” regarding S. aureus having certain protease activities; in addition, once released, the anti-microbial CGA-derived peptides still boost local immunity in dendritic cells and CD14 cells as well in tissues where a high concentration of microbes would justify a sustained release of the host defense peptide. These results indicate that medical use is foreseeable with some synthetic peptides such as CTL or VS-I in the near future, provided that technical and scientific progress enables the perfect impregnation of the implant.

A third issue arose recently: COVID became a significant threat in the hospital as far as its critical forms are concerned. De Lorenzo et al. asserted that CGA concentrations could predict death [36]. They showed that dying COVID patients demonstrated higher CGA levels on admission than survivors. Indeed, in our differently designed study of COVID patients admitted for acute respiratory failure and hypoxemia, admission plasma CGA concentrations instead predicted the occurrence of morbidity rather than mortality, which was better forecast by the CAT/CGA ratio [14]. Our study suggested that the stressing challenge of COVID was probably not hypoxemia itself—in line with its effect in vitro—since matched ICU control patients without hypoxemia did display levels of CGA similar to those of hypoxic COVID patients [37]. Because standard inflammation parameters did also not correlate with either CGA or CAT, we also examined the possibility that long-lasting circulating concentrations of CAT could interfere with systemic inflammation. According to a previous study by our group, such concentrations physiologically boost, through a cell-penetrating-peptide mechanism, the release of pro-inflammatory molecules by PMNs, molecules which are currently recognized as biomarkers of severity in COVID [23]. In addition to this mechanism of inflammation, circulating CAT will also be available to engage in a molecular receptor-linked action on nAChR. There are strong scientific arguments to prove that CAT is a non-competitive inhibitor of this receptor, which provides a new and better comprehension of imbalanced neural regulation of innate immunity in critically ill patients [38]. This activity of CAT will be of major significance regarding all critically ill patients in explaining morbidity linked to the failure to arrive at a proper balance between pro- and anti-inflammatory pathways after life-threatening stress. Indeed, the action of CAT on nAChR provides an explanation not only for the occurrence of care-related infection but also for long-lasting multiple organ failure-associated myopathy [39].

Finally, there are other relevant issues regarding critically ill patients in connection with the in vivo processing of CGA. Among the difficult matters to explore is the involvement of CGA and its derivatives in acute cardiac diseases, whereas its involvement in chronic heart diseases has already been investigated [40,41,42,43,44,45]. Acute cardiac failure is far from exceptional in acute-onset conditions such as shock. No compelling reason exists to exclude a possible role for full-length CGA or selected CGA-derived peptides in transient cardiomyopathy or some severe arrhythmias.

## 7. Conclusions

In recent years, progress has been achieved in comprehending the biological significance of full-length CGA and selective CGA-derived peptides measured in the bloodstream of critically ill patients. These molecules will undoubtedly benefit from specific assessments in larger groups of seriously ill patients with selected clinical phenotypes. Such a prerequisite is mandatory to understand how well-conserved molecules can contribute to understanding pathophysiological conditions that are still unexplained and, therefore, not efficiently treated. For further experiments, we propose testing the implications of CGA and CGA-derived peptides in acute heart diseases and in acute neurological abnormalities that are time-dependently observed in the critically ill. In addition, given the presence of other granins, these molecules will also be investigated in a second step. If our hypotheses turn out to be correct, we believe this will open new insights into the care of the critically ill with multiple organ failure. Linking our efforts in clinical settings and ex vivo experiments will result in better use of endogenous CGA-derived peptides for diagnostic tools and synthetic CGA-derived peptides for implants in the critically ill.

## Data Availability

Not applicable.
